# Tumour progression shows decrease in PD‐L1 expression in matched metastases/primary uveal melanomas

**DOI:** 10.1111/aos.17559

**Published:** 2025-07-24

**Authors:** Maria Chiara Gelmi, Gulçin Gezgin, Ellen Kapiteijn, T. H. Khanh Vu, Martine J. Jager, Robert M. Verdijk

**Affiliations:** ^1^ Department of Ophthalmology Leiden University Medical Center Leiden The Netherlands; ^2^ Department of Medical Oncology Leiden University Medical Center Leiden The Netherlands; ^3^ Department of Pathology Leiden University Medical Center Leiden The Netherlands; ^4^ Department of Pathology, Section Ophthalmic Pathology Erasmus MC University Medical Center Rotterdam The Netherlands

**Keywords:** immune checkpoint, metastases, microenvironment, uveal melanoma

## Abstract

**Purpose:**

Immune checkpoint inhibitors (ICI) have revolutionised the treatment of several malignancies. However, the results of ICI therapy remain unsatisfactory in metastatic uveal melanoma (UM). We analysed the expression of PD1, PD‐L1, T‐cell and macrophage markers in a set of matched primary and metastatic UM in an attempt to better understand the low effectiveness of ICI in metastatic UM.

**Methods:**

Thirty‐two samples (19 metastases and 13 primary UM) were stained for PD‐L1, PD1, CD3, CD4, CD8, CD68, CD163, HLA class I and BAP1. T‐cell markers were scored quantitatively, while PD‐L1, CD68, CD163 and BAP1 were scored semiquantitatively. The immunohistochemical (IHC) scores were compared between all primary and metastatic UM samples and between matched cases.

**Results:**

Both the general and the matched analyses revealed that the IHC scores for PD‐L1 expression on tumour cells were lower in metastatic UM than in primary UM. Conversely, T‐cell markers, including PD1, were significantly higher in UM metastases than primary UM, while macrophages did not show a difference. Metastases with a low HLA Class I expression lacked PD‐L1 and PD1 expression. BAP‐1 loss was associated with increased lymphocytic infiltration.

**Conclusions:**

While UM metastases had higher lymphocytic infiltrates than primary UM, PD‐L1 showed a lower expression in metastases. We believe that the low effectiveness of ICI in the treatment of metastatic UM may be partly explained by the low PD‐L1 expression. We propose that primary tumours may be more responsive to ICI therapy than metastases and could be targeted in a (neo)adjuvant setting for patients at high risk of developing metastases.

## 1 INTRODUCTION

Treatment of metastatic cutaneous melanoma (Hodi et al., 2010; Robert et al., 2015) and other malignancies (such as non‐small cell lung cancer [NSCLC]) (Borghaei et al., 2015; Garon et al., 2015) with immune checkpoint inhibitors (ICIs) has revolutionised the management of patients (Shiravand et al., 2022). Most ICIs act on the PD1/PD‐L1 axis and on CTLA4, but other ICIs exist and are starting to be targeted by new drugs. In metastatic uveal melanoma (UM), however, the results of ICI therapy have been largely unsatisfactory (van der Kooij et al., 2017; Wessely et al., 2020; Wierenga et al., 2019). A good response has only been seen in patients with a tumour mutation in Methyl‐CpG Binding Domain 4 (*MBD4*) (Johansson et al., 2019; Rodrigues et al., 2018; Saint‐Ghislain et al., 2022). This is most likely linked to the higher tumour mutational burden (TMB) in *MBD4*‐mutated UM. In other malignancies, TMB is independently correlated with a positive response to checkpoint inhibitors, but it is usually very low in UM (Goodman et al., 2017; Yarchoan et al., 2017).

Response to ICI therapy may be predicted, mostly using the PD‐L1 expression in the tumour by immunohistochemistry; however, evidence is not always univocal, and it is no longer used for patient selection in cutaneous melanoma (Sadeghi Rad et al., 2020; Topalian et al., 2016).

In prior studies, expression of PD1 and PD‐L1 in UM has mainly been investigated through immunohistochemistry (IHC), both in primary UM and UM metastases (Table S1). PD‐L1 expression varies greatly between studies, due to the differences in scoring methods and sample sizes. The percentage of PD‐L1‐positive tumours varies from 7.8% to 46% in primary UM and from 0% to 23% in UM metastases (Hoefsmit et al., 2020; Javed et al., 2017; Kaunitz et al., 2017; Mariani et al., 2023; Qin et al., 2020; Rossi et al., 2019; Zoroquiain et al., 2018). In a paper by Singh et al., the percentage of PD‐L1 positive cells in primary UM was reported as 62%, but it is not clear if they only scored tumour cells or if they included positive infiltrating immune cells in their scoring (Singh et al., 2021). The presence of PD1‐positive tumour‐infiltrating lymphocytes (TILs) has generally been reported as low in both primary UM and UM metastases, except in one study by Javed et al., which classified cases as positive if at least one PD1+ TIL per high power field was detected (Javed et al., 2017; Mariani et al., 2023; Qin et al., 2020; Rossi et al., 2019; Singh et al., 2021). Few studies have compared the expression of PD‐L1, PD1 and other T‐cell markers in pairs of primary and metastatic UM. Qin et al. analysed 11 pairs of primary and metastatic UM, but could not make generalised statements due to incomplete IHC data (Qin et al., 2020). Mariani et al. based their results on comparative analyses performed both on the total cohort of primary and metastatic UM and on 21 pairs of primary and metastatic UM, indicating lower PD‐L1 expression in metastases compared to primary tumours (Mariani et al., 2023).

To validate and expand the studies described, we analysed the expression of PD‐L1, PD1, T‐cell markers and macrophage markers in a set of matched primary and metastatic UM in an attempt to better understand the low effectiveness of ICI in UM.

We hypothesise that the low effectiveness of ICI in the treatment of metastatic UM may be partly explained by specific adaptations to the immune environment, such as the decreased PD‐L1 expression in UM metastases compared to primary UM.

## 2 METHODS

### 2.1 Patients

In total, 32 samples were included: 13 primary UM and 19 metastases from 17 patients. The 13 primary UM cases were all matched to one or two metastases. The primary samples were collected after enucleation, whereas the metastatic samples were predominantly collected by core biopsy at the time of diagnosis.

### 2.2 IHC staining

Immunohistochemistry was performed using an automated, validated and accredited staining system (Ventana Benchmark ULTRA, Ventana Medical Systems, Tucson, AZ, USA) with an ultraview Universal Alkaline Phosphatase Red Detection Kit (Ventana reference no. 760‐501). Following deparaffinisation and heat‐induced antigen retrieval (CC1 for 32 min), the tissue samples were incubated for 8–44 min with the respective antibodies (Table S2). Counterstain was done by haematoxylin II stain for 12 min and a blue colouring reagent for 8 min according to the manufacturer's instructions (Ventana Benchmark ULTRA, Ventana Medical Systems, Tucson, AZ, USA). For each slide, healthy tonsil tissue was used as a positive control.

### 2.3 HLA class I immunofluorescence staining and scoring

The metastasis samples in this cohort were stained and scored for HLA‐A, HLA‐B/C and β2‐microglobulin with immunofluorescence as previously described by Gezgin et al. (2017). Briefly, sections were stained as described by van Esch et al. (2014) and scored with the scoring system of Ruiter et al. (1998). A score of 0–5 was assigned based on the percentage of positive tumour cells, and a score of 0–3 was assigned based on the staining intensity. The two scores were added up, and samples with a score of 0–2 were classified as negative, those with a score of 3–6 were classified as weak, and samples with a score of 7–8 were classified as positive. The results of these analyses have been published before (Gezgin et al., 2017). Because the scores for all three markers matched in each sample, we defined cases as ‘HLA class I negative’, ‘HLA class I weak’ and ‘HLA class I positive’.

### 2.4 IHC scoring and statistical analyses

PD‐L1 expression was scored by estimating the percentage of tumour cells with positive membrane staining and classifying cases with <5% staining as negative and cases with >5% staining as positive (Javed et al., 2017; Rossi et al., 2019; Zoroquiain et al., 2018). We scored T‐cell markers (PD1, CD3, CD4 and CD8) using the QuPath software by counting the number of positive lymphocytes in 1 mm^2^, as done in previous papers (Qin et al., 2017, 2020). Because core biopsy material of UM metastases may contain both tumour and non‐tumour tissue, we delineated the areas containing only tumour tissue and scored the markers only in the delineated areas. Macrophage markers CD68 and CD163 were scored as low, medium, or high by comparison with example images from Mäkitie et al. (2001) BAP1 was scored as positive if the tumour cell nuclei were positive and was scored as negative otherwise (Kalirai et al., 2014; Koopmans et al., 2014; van Essen et al., 2014). We used the Mann–Whitney *U* test to compare the continuous scores between primary and metastatic UM samples and Fisher's exact test to compare the categorical scores between primary and metastatic UM samples. Statistical analyses were performed using SPSS (IBM Corp), and plots showing the scores in matched samples were created using GraphPad Prism.

### 2.5 Ethical approval

This research was approved by the Scientific Committee of the Ophthalmology Department of the Leiden University Medical Center (project name ‘Onderzoek naar de immuunstatus van choroidale nevi, uveamelanomen en metastasen’, approved under project number 29.1). The research adhered to Dutch law and the tenets of the Declaration of Helsinki (World Medical Association of Declaration 1964; ethical principles for medical research involving human subjects). Following COREON regulations and approval by the scientific committee, patient consent was waived due to the use of left‐over material from diagnostic procedures with no additional burden to the patient.

## 3 RESULTS

We set out to determine the presence of immune‐checkpoint PD1/PD‐L1 and tumour‐infiltrating lymphocytes and macrophages in a set of UM metastases and, if available, the corresponding primary tumour. In total, 32 samples were included: 19 metastases and 13 primary UM. For 15 metastases, the primary tumour was available. Some cases could not be scored for all markers because there was not enough tumour tissue on the slide or because of artefacts (details in Table 1).

**TABLE 1:** List of primary and metastatic UM samples with their immunohistochemical score for BAP1, PD‐L1, PD1, CD3, CD4, CD8, CD68 and CD163.

Patient ID	Sample ID	Prim or met	BAP1	PD‐L1	PD1	CD3	CD4	CD8	CD68	CD163	From Gezgin et al. (2017)			
			Pos/neg	Pos/neg	Nr/mm^2^	Nr/mm^2^	Nr/mm^2^	Nr/mm^2^	Low/medium/high	Low/medium/high	HLA‐A	HLA‐B/C	B2M	HLA class I expression
UM01	S_01	Prim	Neg	1	113	1932	290	846	3	1				
	S_14	Met	Neg	0	19	31	106	3	1	1	0	0	0	Neg
UM02	S_02	Prim	Neg	1	8	61	40	27	3	2				
	S_15	Met	Neg	0	NA^a^	NA^a^	NA^a^	NA^a^	2	2	8	8	8	Pos
	S_16	Met	Neg	0	92	215	120	225	2	1	8	8	8	Pos
UM03	S_03	Prim	Pos	0	17	116	61	19	2	0				
	S_17	Met	Pos	0	73	92	86	68	3	1	3	3	3	Weak
UM04	S_04	Prim	Neg	1	94	191	90	176	3	2				
	S_19	Met	Neg	1	766	2563	893	1270	3	3	8	8	8	Pos
	S_18	Met	Neg	1	1086	3661	2348	2605	NA^a^	NA^a^	8	8	8	Pos
UM05	S_05	Prim	Pos	1	77	NA^c^	268	115	2	1				
	S_20	Met	Pos	0	21	371	109	175	1	1	0	0	0	Neg
UM06	S_06	Prim	Neg	1	139	974	463	860	3	2				
	S_21	Met	Neg	0	876	5184	2625	1955	2	0–1	7	7	7	Pos
UM07	S_07	Prim	Neg	0	10	31	73	24	1	1				
	S_22	Met	NA^b^	NA^b^	128	408	305	190	0	0	NA^c^	0	0	Neg
UM08	S_08	Prim	Neg	1	199	369	264	464	3	2				
	S_23	Met	Neg	0	147	802	341	486	2	2	8	8	8	Pos
UM09	S_09	Prim	Neg	0	107	292	288	226	2	0–1				
	S_24	Met	Neg	0	313	1051	506	735	3	3	8	7	8	Pos
UM10	S_10	Prim	Neg	1	118	377	187	496	3	1				
	S_25	Met	Neg	NA^b^	436	2000	3121	496	3	2	8	8	8	Pos
UM11	S_11	Prim	Neg	1	15	146	44	185	2	1				
	S_26	Met	Neg	0	57	678	NA^c^	484	2	1	5	5	5	Weak
UM12	S_12	Prim	NA^b^	NA^b^	NA^b^	NA^b^	NA^b^	NA^b^	NA^b^	NA^b^				
	S_27	Met	Pos	0	17	409	220	139	2	1	8	8	8	Pos
UM13	S_13	Prim	Pos	0	56	93	NA^c^	50	3	2				
	S_28	Met	Pos	0	NA^a^	NA^a^	673	234	1	1	NA^c^	NA^c^	NA^c^	NA^c^
UM14	S_29	Met	Neg	0	19	45	NA^c^	41	3	1	NA^c^	NA^c^	NA^c^	NA^c^
UM15	S_30	Met	Neg	NA^b^	236	1797	573	2084	3	2	8	8	8	Pos
UM16	S_31	Met	Pos	1	322	1182	413	1149	2	2	8	8	8	Pos
UM17	S_32	Met	Neg	0	555	726	235	581	2	1	8	8	8	Pos

Abbreviation: B2M, beta 2 microglobulin.

^a^ Unsure of tumour area.

^b^ No tumour/insufficient tumour on slide.

^c^ Artefact/unsure which cells are positive.

### 3.1 IHC scoring for PD‐L1, T cells/PD1, macrophages and BAP1

We stained all primary and metastatic tumour samples for PD‐L1, PD1, T‐cell markers CD3, CD4 and CD8, macrophage markers CD68 and CD163 and BAP1. Figure S1 shows the IHC stainings in a pair of BAP1‐negative primary and metastatic UM.

**FIGURE 1:**
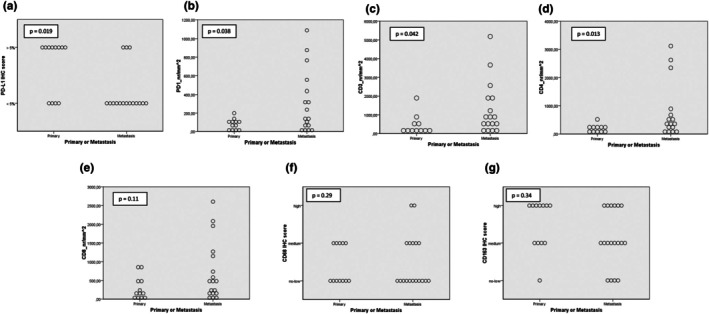
Dot plots showing PD‐L1, PD1, other T‐cell markers and macrophage marker scores in all primary and metastatic UM samples. (a) PD‐L1. (b) PD1. (c) CD3. (d) C4. (e) CD8. (f) CD68. (g) CD163. Most of the primary UM samples have low IHC scores for PD1 and T‐cell markers, while UM metastatic samples show higher variability.

**FIGURE 2:**
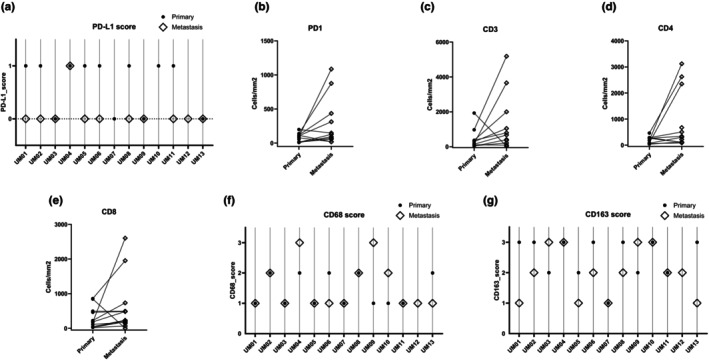
PD‐L1, PD1, other T‐cell markers and macrophage marker scores in matched primary and metastatic UM. (a) PD‐L1. (b) PD1. (c) CD3. (d) CD4. (e) CD8. (f) CD68. (g) CD163. (a, f, g) Each vertical line corresponds to one pair of primary and metastatic UM. (b–e) Line plots in which each line corresponds to one pair of primary and metastatic UM. Metastases are more frequently PD‐L1 negative than primary UM, while there is no difference in macrophage score between primary UM and UM metastases.

We first compared the IHC score of all markers between primary and metastatic UM samples, considering all samples (Figure 1 and Table 2). We then explored the differences in infiltrate markers between matched metastases and primary samples (Figure 2 and Table 3). To account for the magnitude of change in T‐cell markers, for each pair, we calculated the ratio of the metastasis score over the primary score (M/P) and presented the median of these ratios in Table 3.

**TABLE 2:** Distribution of infiltrate markers in all primary UM and UM metastasis samples.

	Primary UM (*N* = 13)^c^	UM metastasis (*N* = 19)^c^	*p* Value
PD‐L1			
Negative	4 (33%)	13 (81%)	0.019^b^
Positive	8 (67%)	3 (19%)	
PD1			
Median (range)	85.32 (8.00–198.87)	147.03 (16.99–1086.15)	0.038^a^
CD3			
Median (range)	190.93 (30.81–1932.28)	726.37 (30.99–5184.09)	0.042^a^
CD4			
Median (range)	186.93 (39.72–462.56)	376.84 (85.97–3120.51)	0.013^a^
CD8			
Median (range)	180.24 (18.99–859.84)	485.16 (3.00–2605.02)	0.113^a^
CD68			
Low	7 (58%)	11 (61%)	0.29^b^
Medium	5 (42%)	5 (28%)	
High	0	2 (11%)	
CD163			
Low	1 (8%)	4 (22%)	0.34^b^
Medium	4 (33%)	8 (44%)	
High	7 (58%)	6 (33%)	

^a^ Mann–Whitney *U* test.

^b^ Fisher's exact test.

^c^ Some cases could not be scored: refer to Table S1.

**TABLE 3:** Comparison of PD‐L1, PD1, other T‐cell markers and macrophage markers in matched primary and metastatic UM samples.

a: PD‐L1 expression in matched primary UM and UM metastases			
	Cases with metastasis > primary	Cases with primary > metastasis	Cases with no change
PD‐L1	0	6	4

b: Distribution of T‐cell markers in matched primary UM and UM metastases			
	Number of cases with metastasis > primary	Number of cases with primary > metastasis	Ratio metastasis/primary (median)
PD1	8	3	3.80
CD3	8	2	4.12
CD4	8	2	2.39
CD8	10	2	2.94

c: Macrophage markers in matched primary UM and UM metastases			
	Cases with metastasis > primary	Cases with primary > metastasis	Cases with no change
CD68	3	2	7
CD163	2	6	4

As shown in Figure 1, all markers showed quite some variation in the group of the primary tumours as well as in the group of metastases. Across all samples, metastases were less frequently PD‐L1 positive compared to primary UM samples (19% PD‐L1 positive cases in metastases (*n* = 3/19) vs. 67% PD‐L1 positive cases among primary tumours (*n* = 8/13)) (Table 2 and Figure 1a). The plot with matched primary and metastatic samples in Figure 2a and Table 3 confirms this trend: none of the matched cases showed a PD‐L1 score higher in metastasis than in its primary tumour, 6 cases had a primary score higher than the metastasis score and 4 cases did not show any difference (Figure 2a).

There was a good correlation between all T‐cell markers (PD1, CD3, CD4, CD8) (Figure S2). When considering all samples, the scores for PD1, CD3, CD4, were significantly higher in the metastatic samples compared to the primary UM samples (PD1: *p* = 0.04; CD3: *p* = 0.04; CD4: *p* = 0.01), while the difference was not significant for the CD8 score (Table 2 and Figure 1b–e). For the majority of the pairs, the T‐cell markers were (much) higher in the metastasis than in the primary samples (PD1, CD3, CD4 in 8 cases, CD8 in 10 cases), while a few pairs had higher scores in the primary than in the metastasis samples (PD1 in 3 cases, CD3, CD4 in 2 cases, CD8 in 2 cases) (Table 3 and Figure 2b–e). All T‐cell markers, including PD1, showed median M/P ratio >1.0, thus confirming that metastatic samples had a more prominent T‐cell infiltrate compared to the matched primary samples (Table 3).

We did not see any relevant difference in macrophage markers CD68 and CD163 between primary UM and metastases (Tables 2 and 3 and Figures 1f,g and 2f,g).

We next checked how infiltrate markers related to the IHC BAP1 status of our samples (Listed in Table 1). Of the 13 primary UM samples, one could not be scored because there was no tumour visible on the slide, 9 (69%) were BAP1 negative and 3 (23%) were BAP1 positive. Of the 19 UM metastasis samples, one could not be scored because of insufficient tumour material, 13 (68%) were BAP1 negative and 5 (26%) were BAP1 positive. All pairs showed a match in BAP1 expression between the primary and metastatic samples. Figure S3a shows that most of the PD‐L1‐positive samples were BAP1‐negative primary UM. As shown in Figure S3, the IHC scores for T‐cell markers were low in all BAP1‐positive cases, both in primary tumours as well as in metastases. In the BAP1‐negative cases, the scores for T‐cell markers were more variable, especially in metastases, which had higher scores compared to primary BAP1‐negative samples (Figure S3b–e). The scores for macrophage markers CD68 and CD163 were higher in BAP1‐negative compared to BAP1‐positive cases, but did not show clear differences between BAP1‐negative primary and metastases UM (Figure S3f,g).

### 3.2 HLA class I expression in UM metastases

We compared the presence of infiltrating leukocytes and PDL1 with HLA Class I expression on metastases, as previously determined (Gezgin et al., 2017). Of the 19 metastases in this cohort, two could not be scored for HLA Class I. Of the remaining 17, three were classified as negative, two as weak and 12 as positive. We combined the ‘negative’ and ‘weak’ groups and compared the IHC scores between ‘HLA class I negative‐weak’ and ‘HLA class I positive’ cases (Figure S4). Metastases with a negative/weak HLA expression lacked PD‐L1 expression and showed very few infiltrating lymphocytes. PD‐L1 expression was variable in metastases with a high HLA class I expression.

## 4 DISCUSSION

We analysed the expression of PD‐L1, T‐cell markers (PD1, CD3, CD4, CD8), macrophage markers (CD68, CD163) and BAP1 in a cohort of matched primary and metastatic UM samples. PD‐L1 expression on tumour cells was lower in metastatic UM samples than in primary UM. The IHC scores for CD3, CD4 and PD1 were significantly higher in UM metastases than in primary UM. CD8 expression showed a similar tendency but did not reach significance. We did not find any difference between primary UM and metastases in macrophage markers.

### 4.1 Immune infiltrate in primary and metastatic UM

The immune infiltrate of primary UM is made mainly of macrophages and tumour‐infiltrating lymphocytes (TILs), with CD8+ T cells being more prominent than CD4+ T cells (Bronkhorst et al., 2011, 2012; Hurdogan et al., 2022; Lagouros et al., 2009; Maat et al., 2008; Mäkitie et al., 2001; Qin et al., 2020). A previous study on primary UM samples already showed good correlation between different T‐cell markers (CD3, CD4 and CD8), suggesting that multiple T‐cell subtypes increase in tumours with an inflammatory phenotype (de Waard‐Siebinga et al., 1996). We and others have shown that the presence of both macrophages and TILs is associated with negative prognostic factors and with worse survival (Bronkhorst et al., 2011, 2012; Lagouros et al., 2009; Maat et al., 2008; Zoroquiain et al., 2018). There is evidence suggesting that the microenvironment of primary UM is immunosuppressive: several authors have shown most of the macrophages to be M2 and a considerable portion of the T cells to be Tregs expressing FoxP3, although not all reports are univocal (Bronkhorst et al., 2012; Figueiredo et al., 2020; Hurdogan et al., 2022; Lagouros et al., 2009; Qin et al., 2020; Triozzi et al., 2019). Similarly, the inflammatory infiltrate in UM metastases consists, for the most part, of macrophages, followed by T cells (Krishna et al., 2017; Qin et al., 2020; Tosi et al., 2021). A study involving 21 patients with metastatic UM showed that cases that achieved disease control with systemic or targeted therapies had higher CD8+ T cells, higher CD8/CD4 ratio, lower CD4+ FoxP3+ cells and lower CD163+ cells compared to cases with disease progression (Tosi et al., 2021). One group recently performed immunogenomic analyses of 100 UM metastases from 84 patients and showed that over half of the metastases had TILs that were tumour‐reactive but quiescent (Leonard‐Murali et al., 2024). Because these TILs showed high ex‐vivo expansion, the authors suggest that the quiescent state is not induced by an intrinsic proliferative defect, but rather by the microenvironment (Leonard‐Murali et al., 2024). Few studies have compared primary UM and UM metastases and revealed that metastases usually have a more prominent inflammatory infiltrate compared to primary UM (Mariani et al., 2023; Qin et al., 2020). Interestingly, Qin et al. found no difference in the inflammatory infiltrate between biopsies of treatment‐naïve metastases and those of treated metastases or between metastases treated with immunotherapy vs. other therapies (Qin et al., 2020). They also analysed the expression of PD‐L1 and found no differences between treatment‐naïve and treated metastases.

### 4.2 PD‐L1 as (neo)adjuvant therapy

In cutaneous melanoma and other cancers, a few markers can be used to predict response to anti‐PD1 therapy: PD‐L1 expression, tumour mutational burden and location of metastases. However, no testing is required in cutaneous melanoma because the response rate is high anyway. In UM, all of these markers are not favourable. As mentioned earlier, the expression of PD‐L1 in UM is lower than in cutaneous melanoma, and previous papers by Qin et al. and by Mariani et al. reported PD‐L1 expression to be even lower in UM metastases than in primary UM samples. PD‐L1 expression was lower in metastases than in primary UM samples in our cohort as well, both in the general analysis and the paired analysis, in contrast with the lymphocyte markers. The lower expression of PD‐L1 in metastases compared to the primary UM, evident both in general and in matched analyses, could be one of the reasons why immune checkpoint inhibitors are not effective in metastatic UM. We propose that primary tumours may be more responsive to immune checkpoint therapy than metastases, and that these agents could be used in a neoadjuvant setting in patients who carry a high risk of developing metastases. We are aware of the risk of adverse events that come with the administration of immune checkpoint inhibitors and that it would be unethical to administer such therapies when they are not needed. However, the careful and targeted selection of only the patient group with the highest risk of metastases or those with *MBD4* mutations could shift the risk–benefit balance towards a beneficial role. This would require obtaining tumour tissue to analyse by biopsy, which is not yet common practice in many centres but would be extremely important in case an effective (neo)adjuvant therapy is discovered. A similar rationale prompted two studies using dendritic cell vaccination in patients with high‐risk UM. Trial NCT00929019 was terminated because of slow patient accrual, whereas trial NCT01983748 is still active, but no results have been published yet. Moreover, the ATOM trial, planned to start in October 2024, will use Tebentafusp (a bispecific protein with a T‐cell receptor targeting gp100 and an anti‐CD3 antibody) versus observation in the adjuvant setting of patients with high‐risk UM and who carry HLA‐A*02:01 (NCT06246149).

It is known that the tumour mutational burden of UM is among the lowest of all cancers (Yarchoan et al., 2017). A parallel can be drawn between UM and glioma. Similarly to UM, gliomas and glioblastomas have a low tumour mutational burden and an immunosuppressive microenvironment (Yarchoan et al., 2017). Like the eye, the immune environment of the brain is privileged and contains several elements that protect this organ from inflammation, such as the blood‐brain barrier. Interestingly, gliomas and glioblastomas very rarely give rise to distant metastases. As for immunotherapy, only a small subset of the patients with gliomas and glioblastomas respond well to immune checkpoint inhibitors, while in general, these tumours are not responsive (Arrieta et al., 2023; Wang et al., 2024). Therefore, efforts should be made to correctly identify the features that characterise the patients who may benefit from this type of therapy, such as testing for mismatch repair gene anomalies or tumour mutational burden.

One further element that does not bode in favour of the use of ICIs in UM is the liver tropism of UM metastases. Studies on other cancers have shown that patients with liver metastases have limited benefit from immunotherapy (Yu et al., 2021), possibly due to an immunosuppressive environment in liver metastases. This issue has not been studied extensively in UM, but some reports with minimal response to immunotherapy support these findings (Karydis et al., 2016; Koch et al., 2021; Pelster et al., 2021; Piulats et al., 2021).

### 4.3 Other potential immune checkpoints to target

As summarised in a recent review by Wang et al., expression of PD1 and PDL1 is not the only determinant of response to ICI, but multiple mechanisms can be responsible for resistance to ICIs, including T‐cell desertification and exclusion (Wang et al., 2023). Among other ICIs, lymphocyte‐activation gene 3 (LAG‐3), T‐cell immunoglobulin and mucin domain‐containing protein 3 (TIM‐3) and T‐cell immunoreceptor with Ig and ITIM domains (TIGIT) seem to be potentially relevant in UM (Durante et al., 2020; Souri et al., 2021; Stalhammar et al., 2019). A phase II/III trial showed higher response rates and longer PFS in patients receiving a combination of anti‐LAG‐3 antibodies and anti‐PD1 antibodies compared to patients treated with anti‐PD1 antibodies alone in advanced unresectable or metastatic cutaneous melanoma (Tawbi et al., 2022). This led to FDA approval of this treatment option in cutaneous melanoma. A single‐arm phase II trial studying this drug combination is currently underway in patients with metastatic UM as well (NCT04552223) (Lutzky et al., 2021). Initial clinical studies using anti‐TIM‐3 antibodies (NCT03652077) and a combination of anti‐TIGIT and anti‐PD‐L1 antibodies (NCT05483400) in advanced metastatic solid tumours are being performed. Similarly to our results with PD‐L1, two other immune checkpoints (TIGIT and IDO) have been reported to have higher expression in primary UM than in metastases in an IHC study by Stalhammar et al. (2019). Since low expression of potential targets may limit the effectiveness of therapies, we believe it is extremely important to keep studying the expression and distribution of ICIs on UM metastases, in addition to primary UM.

In this study, we show that PD‐L1 expression is downregulated in UM metastases compared to (matched) primary tumours and is associated with an increase in TIL. Metastases with a negative/weak HLA expression lacked PD‐L1 expression and showed very few infiltrating lymphocytes. This indicates that one should not only check for the expression of immune checkpoints but also HLA class I expression levels on tumour cells to select patients for immune therapies. We believe that the low effectiveness of ICI in the treatment of metastatic UM may be partly explained by this difference in PD‐L1 expression between primary UM and UM metastases. We propose that primary tumours may therefore be more responsive to ICI therapy than metastases and that these agents could be used in a (neo)adjuvant setting in patients who carry a high risk of developing metastases. A primary tumour biopsy would enable careful and targeted selection of the non‐enucleated patient group with the highest risk of metastases to be included in such trials.

## FUNDING INFORMATION

M. C. Gelmi was funded by the Bontius Foundation, Oogfonds, the Sam Fund, the Leiden University Fund, P.A. Jager‐van Gelder Fund, the Blinden‐Penning foundation and ASROO (Associazione Scientifica Retinoblastoma ed. Oncologia Oculare).

## Supporting information


Table S1

Table S2

Figure S1

Figure S2

Figure S3

Figure S4
