# Supplementary material for: Tumour progression shows decrease in PD‐L1 expression in matched metastases/primary uveal melanomas
Source: Acta Ophthalmol. 2025 Jul 24;104(2):164–72. doi: 10.1111/aos.17559 (PMC12888952; doi:10.1111/aos.17559)
Supplement: Supplementary file 1 — Table S1 [file AOS-104-164-s006.pdf]

Supplemental Table S1: PD-L1 and PD1 immunohistochemical scoring in primary and metastatic uveal melanoma in the literature.							
	PD-L1			PD1			Notes
	Method	Primary	Metastasis	Method	Primary	Metastasis	
Kaunitz 2017 (ref. 16)	+ if > 5% of tumour cells had PD-L1 membrane staining	10% (total 103)		Not performed			101 prim, 2 met
Javed 2017 (ref. 20)	+ if ≥ 5% of tumour cells had PD-L1 membrane staining with 2/3+ intensity		5% (4/78)	In hotspots: + if ≥ 1 PD1+ TIL per hpf		51% (23/45)	
Zoroquian 2018 (ref. 19)	+ if ≥ 5% of tumour cells had PD-L1 membrane only or membrane and cytoplasmic staining	46% (total 67)		Not performed			PDL1+ 50% in non metastatic cases, 40% in metastatic cases
Rossi 2019 (ref. 22)	+ if ≥ 5% of tumour cells had PD-L1 membrane only or membrane and cytoplasmic staining	7.8% (total 14)	10% (total 10)	Not mentioned	0 (total 14)	10% (total 10)	Not predictive of treatment response
Hoefsmit 2020 (ref. 17)	% of PD-L1+ cells scored in 3 levels: < 1%, 1-50%, > 50%. + if > 1%.		23% (11/47)	Not performed			
Qin 2020 (ref. 18)	+ if any PD-L1 + cell	34% (total 27)	0 (total 31)	In 1-5 areas of 1 mm^2 --> nr of PD1+ cells/area --> normalised as counts/mm^2	Low	Low (no diff naïve vs treated)	-PD1 correlated with inflamm infiltrate<br>-IFNg higher in responders
Singh 2021 (ref. 23)	score: intensity x % of PD-L1 + cells. + if IHC score > 4.	62% (44/71)		score: intensity x % of PD1 + cells. + if IHC score > 4.	42% (30/71)		
Mariani 2023 (ref. 21)	% of tumour and immune cells with PD-L1 membrane staining: 0 = absent; 1 = <10%; 2 = 10-50%, 3 = > 50%.	Mean score 9.4% (total 28)	Mean score 3.2% (total 62)	% of area with PD1+ cells: 0 absent; 1 rare (<5% of tumoral/peritumoral area); 2: 5-50%	Mean score <5% (total 28)	Mean score <5% (total 62)	Intratumoural vs peritumoural different
