# Supplementary material for: Tumour progression shows decrease in PD‐L1 expression in matched metastases/primary uveal melanomas
Source: Acta Ophthalmol. 2025 Jul 24;104(2):164–72. doi: 10.1111/aos.17559 (PMC12888952; doi:10.1111/aos.17559)
Supplement: Supplementary file 2 — Table S2 [file AOS-104-164-s004.pdf]

| Supplemental Table S2: list of antibodies used for immunohistochemistry staining | | | | | | | |
|---|---|---|---|---|---|---|---|
| Antibody | Cell type | Species | Conc. | Company | Clone | Procedure | Incubation |
| BAP1 | n.a. | Mouse | 1/50 | Bio-SB | BSB-109 | Ultraview CC1 64′ | 60 min |
| CD3 | T-cell | Rabbit | 1.4 µg/ml | Ventana | 2GV6 | Optiview CC1 32′ | 32 min |
| CD4 | T-helper cell | Rabbit | 2.5 µg/ml | Ventana | SP35 | Ultraview CC1 64′ | 8 min |
| CD8 | Cytotoxic T cell | Rabbit | 0.35 µg/ml | Ventana | SP57 | Optiview CC1 16′ | 32 min |
| CD20 | B cell | Mouse | 0.3 µg/ml | Ventana | L26 | Ultraview CC1 64′ | 44 min |
| CD68 | M1-macrophage | Mouse | 0.4 µg/ml | Ventana | KP1 | Optiview CC1 16′ | 8 min |
| CD163 | M2-macrophage | Mouse | 0.78 µg/ml | Ventana | MRQ-26 | Optiview CC1 32′ | 32 min |
| PD-L1 | n.a. | Rabbit | 7 µg/ml | Ventana | SP142 | Optiview CC1 64′ | 32 min |
| PD1 | n.a. | Mouse | 5 µg/ml | Cell Marque | NAT105 | Optiview CC1 64′ | 16 min |
