# Supplementary material for: Tumour progression shows decrease in PD‐L1 expression in matched metastases/primary uveal melanomas
Source: Acta Ophthalmol. 2025 Jul 24;104(2):164–72. doi: 10.1111/aos.17559 (PMC12888952; doi:10.1111/aos.17559)
Supplement: Supplementary file 3 — Figure S1 [file AOS-104-164-s001.pdf]

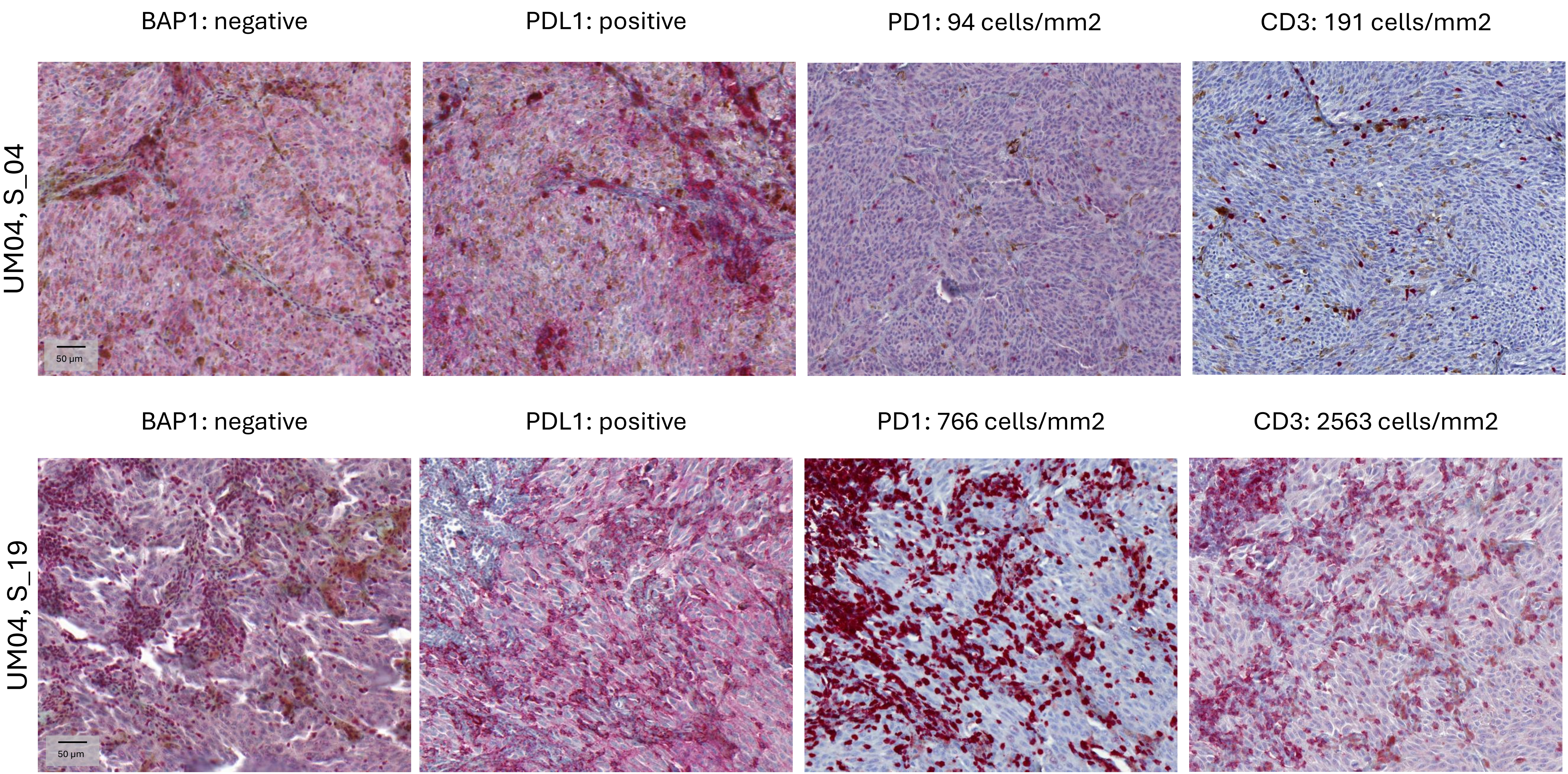
BAP1: negative
PDL1: positive
PD1: 94 cells/mm2
CD3: 191 cells/mm2
UM04, S_04
50 µm
BAP1: negative
PDL1: positive
PD1: 766 cells/mm2
CD3: 2563 cells/mm2
UM04, S_19
50 µm
