# Supplementary figures and images for: Tumour progression shows decrease in PD‐L1 expression in matched metastases/primary uveal melanomas
Source: Acta Ophthalmol. 2025 Jul 24;104(2):164–72. doi: 10.1111/aos.17559 (PMC12888952; doi:10.1111/aos.17559)

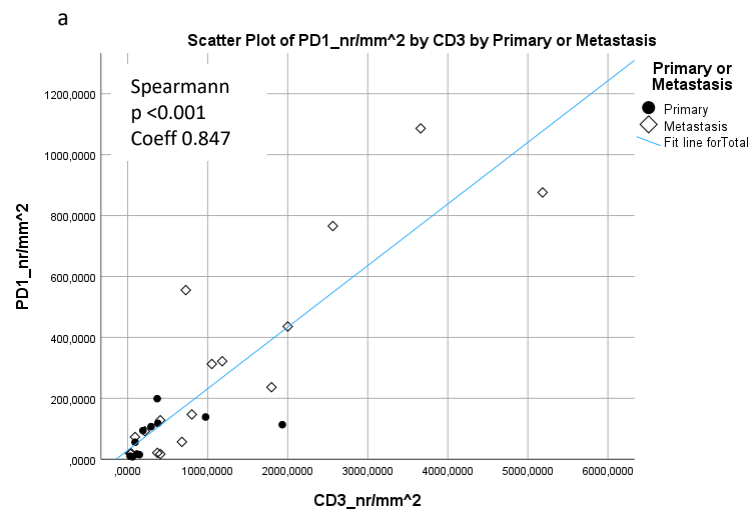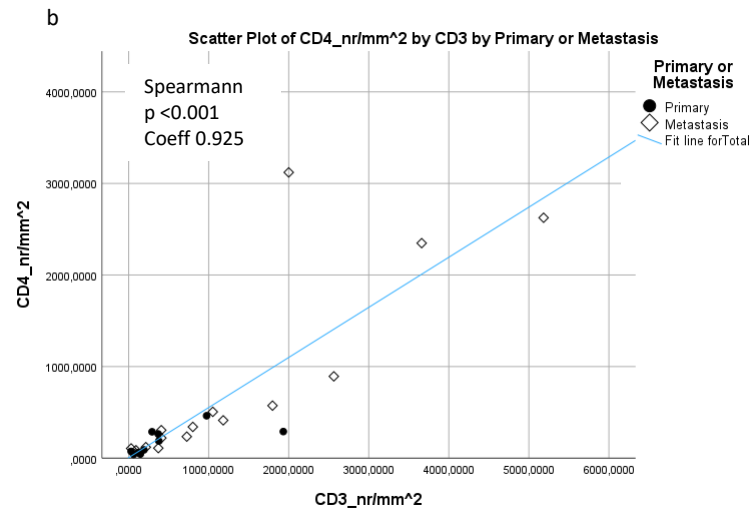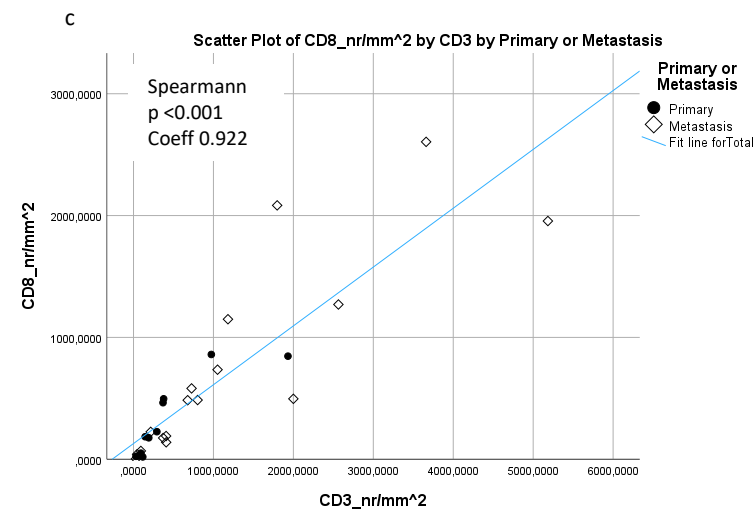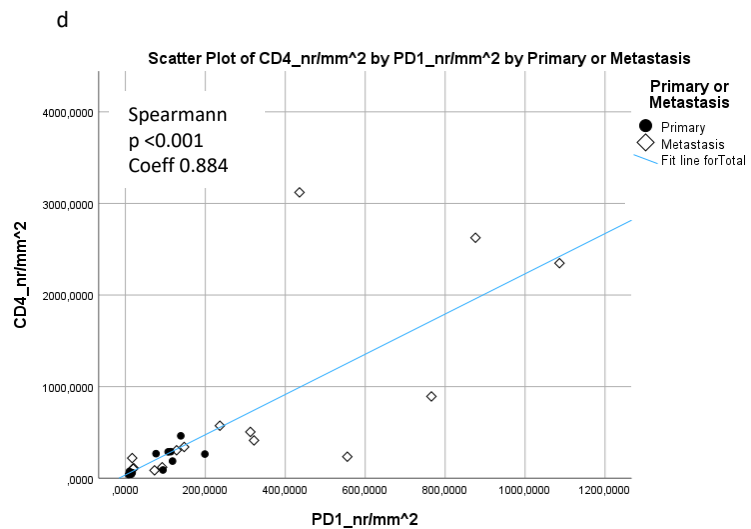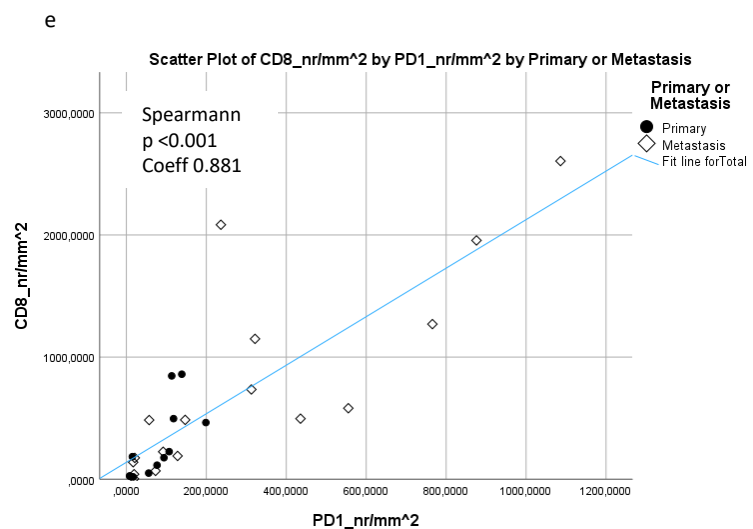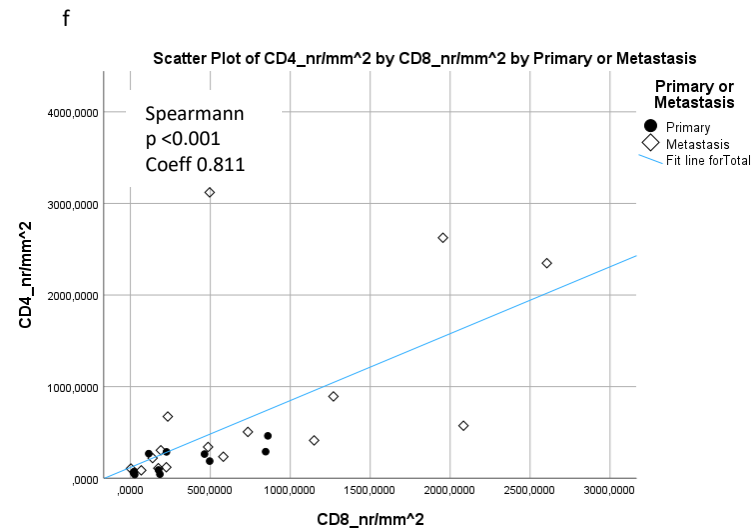

Supplement: Supplementary file 4 — Figure S2 [file AOS-104-164-s002.pdf]

a

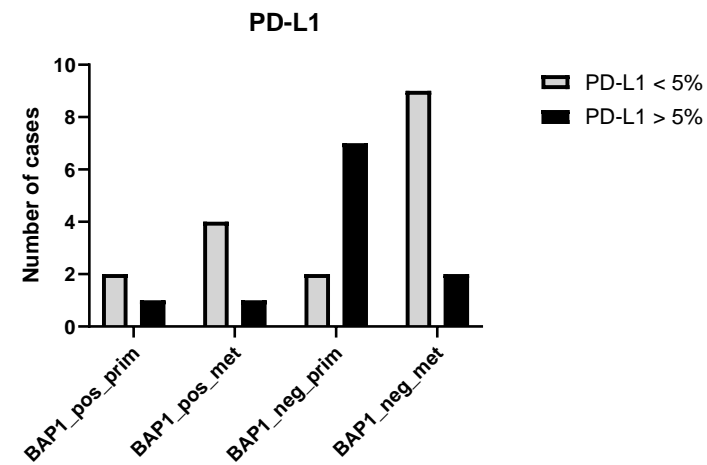

b

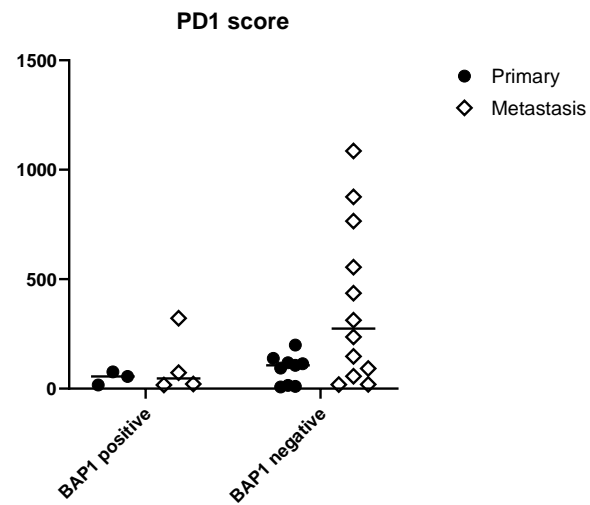

c

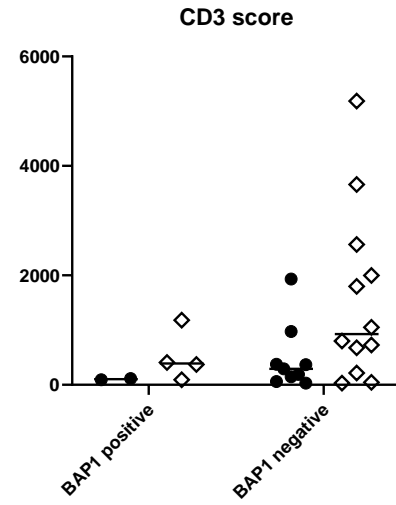

d

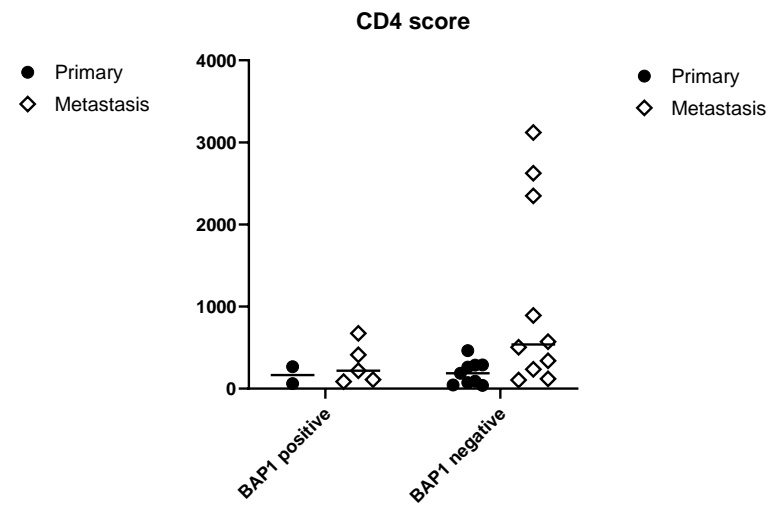

e

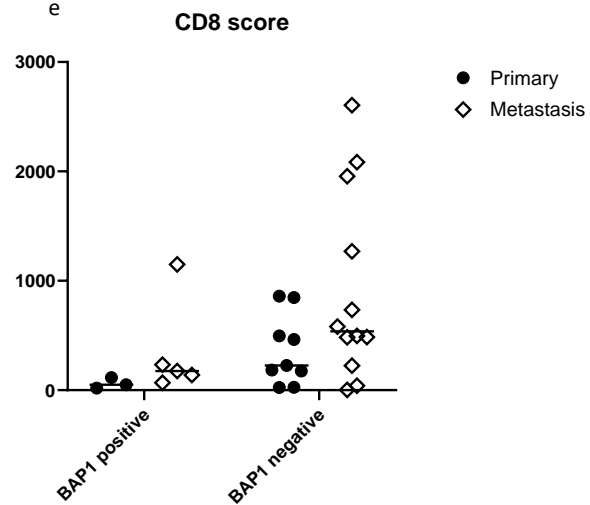

f

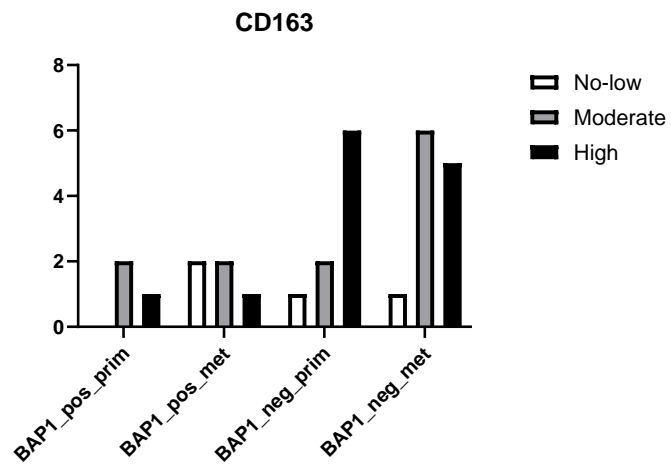

g

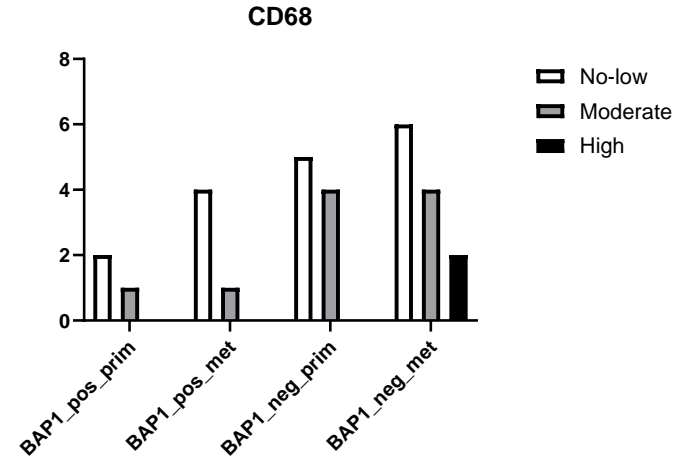

Supplement: Supplementary file 5 — Figure S3 [file AOS-104-164-s005.pdf]

a

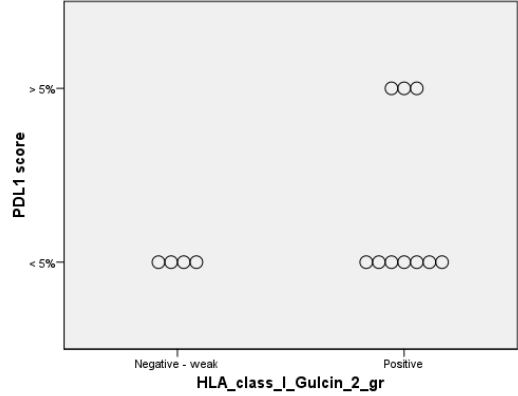

b

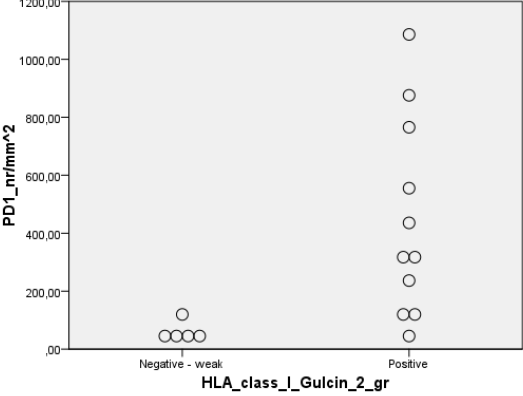

c

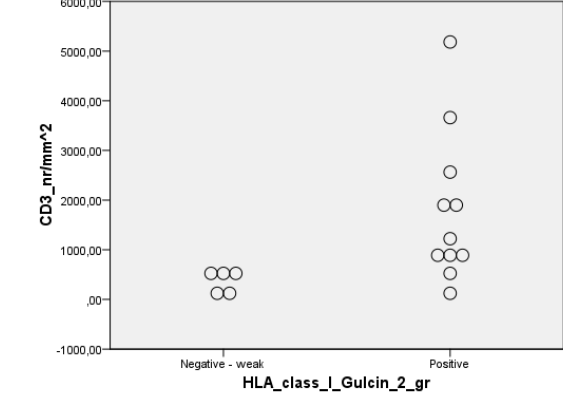

d

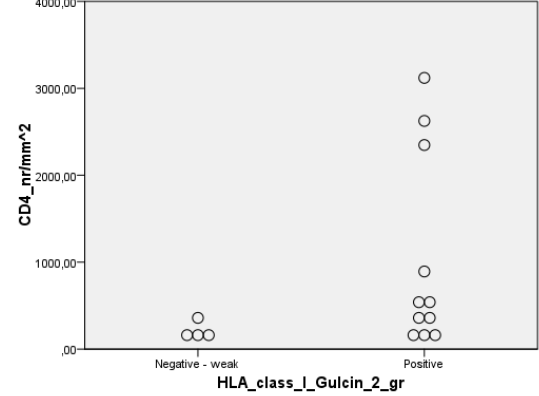

e

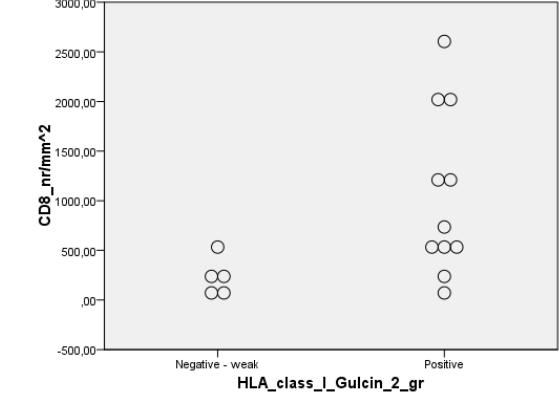

f

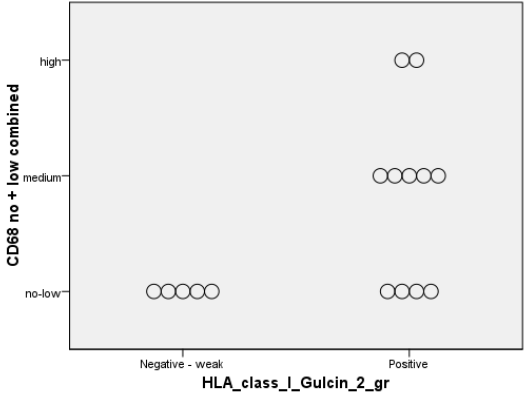

g

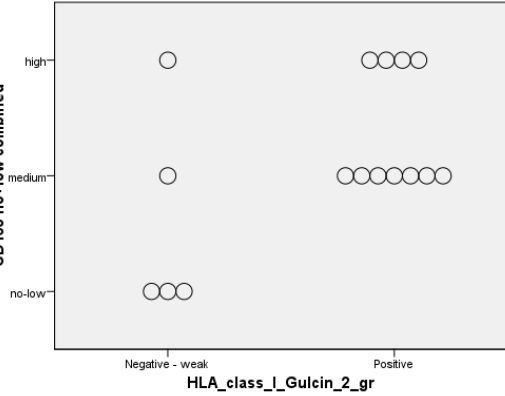

Supplement: Supplementary file 6 — Figure S4 [file AOS-104-164-s003.pdf]
